# Brief report of the construction of infectious DNA clones of South African genetic variants of grapevine virus A and grapevine virus B

**DOI:** 10.1186/s40064-015-1517-2

**Published:** 2015-11-26

**Authors:** D. E. Goszczynski

**Affiliations:** Plant Protection Research Institute, Agricultural Research Council, Private Bag X134, Queenswood, Pretoria, 0121 South Africa

**Keywords:** Grapevine virus A, Grapevine virus B, Genetic variants, Infectious DNA clones

## Abstract

**Background:**

Recent research results strongly suggest that certain genetic variants of grapevine virus A (GVA) and grapevine virus B (GVB), two members of the *Vitivirus* genus of the family *Betaflexiviridae*, are the cause of Shiraz disease and corky bark disease of grapevines in South Africa, respectively. To investigate this hypothesis, work was undertaken to construct DNA clones of these viruses.

**Findings and conclusions:**

Biologically viable and stable DNA clones of genetic variants of GVA and GVB B from South Africa were constructed. The clones share 76.3, 73.2 and 85.2, 77.6 % nt sequence similarity with corresponding clones constructed in Italy and Israel. The results suggest that a derivative of a mini binary vector pCB302 is superior to pCAMBIA1305.1 for the construction of infectious and stable DNA clones of vitiviruses. Successful construction of such DNA clones of GVA and GVB reported in this study is a clear step towards fulfilling Koch’s 3rd postulate in investigating the aetiology of Shiraz disease and corky bark disease.

## Background

**Fig. 1:**
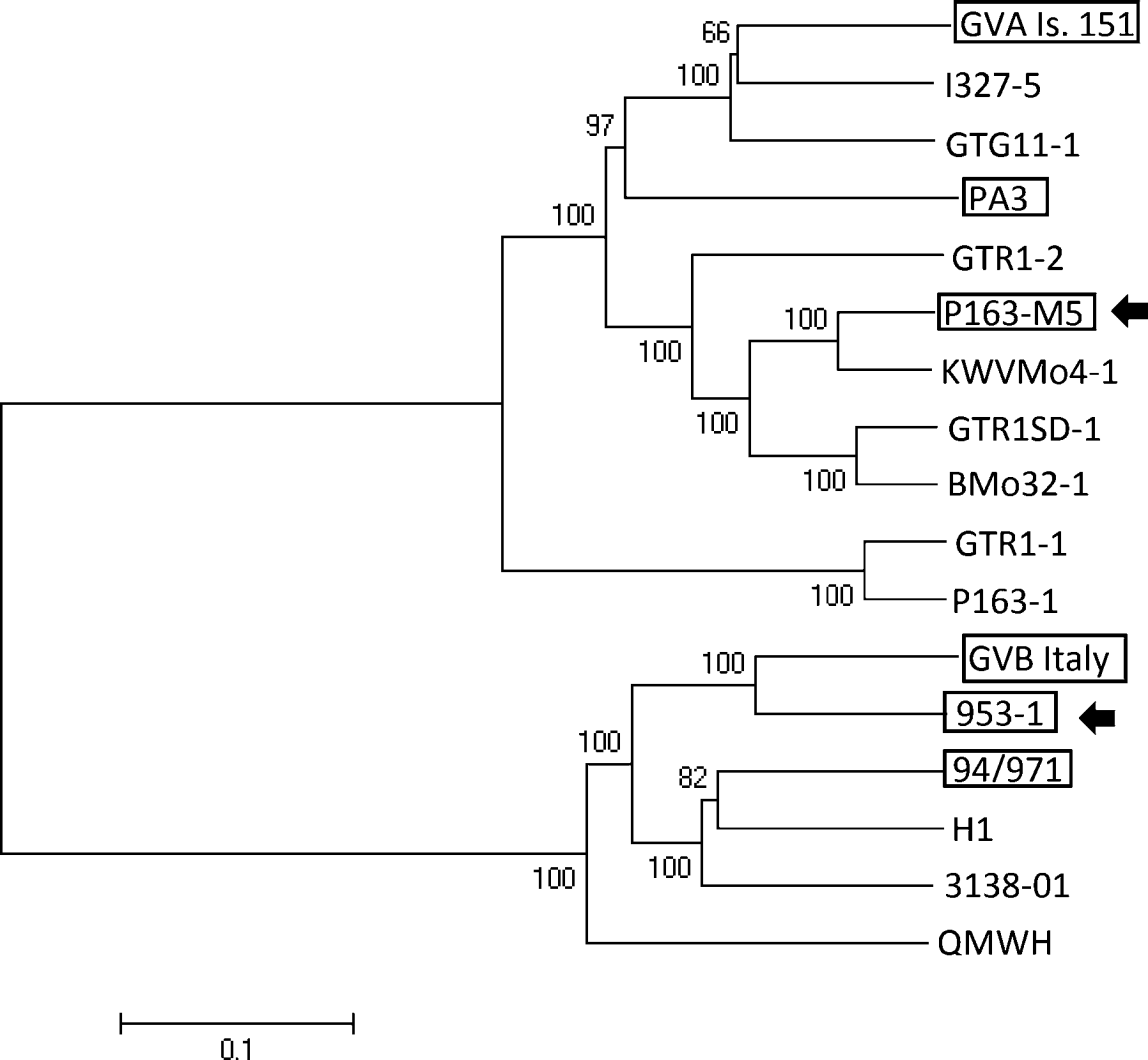
Phylogenetic tree constructed using full genome sequences of various genetic variants GVA and GVB deposited in the GenBank/EMBL database, illustrating the position of the GVA P163-M5 and GVB 953-1 (*arrows*) used in this study. The virus variants, from which biologically active DNA copies were constructed, are boxed. The phylogenetic tree was constructed with the neighbor-joining method (Saitou and Nei 1987) based on evolutionary distances calculated according to the method of Kimura (1980) using MEGA version 4 (Tamura et al. 2007). A bootstrap analysis of data, based on 1000 permutations, was used to assess the statistical confidence of the topology of the tree. The following full genome GenBank/EMBL sequence data was used: GVA isolates 151 (NC003604), I327-5 (KC962564), GTG11-1 (DQ855084), PA3 (AF007515), GTR1-2 (DQ855086), KWVMo4-1 (DQ855083), GTR1SD-1 (DQ855081), BMo32-1 (DQ855087), GTR1-1 (DQ787959) and P163-1 (DQ855088), and GVB isolates Italy (NC003602), 94/971 (EF583906), H1 (GU733707), 3138-01 (JX13897) and QMWH (KF700375)

Grapevine virus A (GVA) and grapevine virus B (GVB) are members of the *Vitivirus* genus of the family Betaflexiviridae (Martelli et al. 2007; Anonym 2012). The genomes, positive strand ssRNA, 7351–7471 nt and 7599–7601 nt, excluding the poly A tails at the 3′ terminus, are organised in five open reading frames (ORF), which are flanked at the 5′ and 3′ terminal parts by 86 nt and 68 nt, and 48 nt and 147 nt not translated sequences (Minafra et al. 1997; Sardarelli et al. 1996). ORF1, 3, 4 and 5 encodes RNA dependent RNA polymerase, movement protein, capsid protein and RNA-binding protein, respectively. The function of the protein encoded by ORF2 is not known (Minafra et al. 1997; Sardarelli et al. 1996; Galiakparov et al. 2003). The viruses are extensively genetically heterogenic (Shi et al. 2004; Goszczynski and Jooste 2003a; Murolo et al. 2008; Voncina et al. 2011; Wang et al. 2011, 2012; Alabi et al. 2014). Genetic variants of these viruses, whose full genome sequences are deposited in the GenBank (Fig. 1), share 70.4–86.0 % and 75.4–85.2 % nt similarity. GVA and GVB are able to multiply in herbaceous hosts such as various species of *Nicotiana* ssp. (Boscia et al. 1997).

Although GVA and GVB have already been reported in 1980 (Conti et al. 1980) and 1993 (Boscia et al. 1993) respectively, and are common in vineyards world-wide, their pathogenicity to grapevines remains unknown. Research results suggest that GVA and GVB may cause two of the four rugose wood [RW] diseases of grapevines, Kober stem grooving (KSG) (Boscia et al. 1997; Garau et al. 1994; Minafra 2000) and corky bark (CB) (Boscia et al. 1993, 1997; Bonavia et al. 1996; Minafra 2000), respectively. Abnormal development of cambium cells causing modification of wood cylinder and bark in canes of infected plants is a common feature of RW diseases (Minafra 2000). KSG and CB diseases only induce strong symptoms on few grapevines like Kober 5BB and LN33 hybrids. Despite this limited range of grapevines susceptible to KSG and CB diseases there is common belief that these diseases negatively influence graft take of all grapevine cultivars to rootstocks that leads to reduced vigor of plants, and ultimately result in lower productivity and longevity of vineyards (Boscia et al. 1997; Minafra 2000). Recent results also revealed that certain genetic variants of GVA are associated with Shiraz disease (SD), which is highly destructive for grapevines cv. Shiraz and Merlot in South Africa (Goszczynski and Jooste 2003b; Goszczynski 2007; Goszczynski et al. 2008). Canes of SD-affected grapevines never mature and the plants usually die in 3–5 years. Despite the association of GVA and GVB with KSG, SD and CB, the aetiologies of these diseases are still not clear since grapevines are usually infected with a mixture of different virus species. Genetically uniform populations of GVA and GVB needed to fulfill Koch’s 3rd postulate—isolation of a pathogen and re-infection of a host followed by the development of disease symptoms, can be obtained relatively quickly by the construction of the DNA clones of these viruses. Although the biologically active DNA clones of various genetic variants of GVA and GVB have already been constructed in laboratories in Italy (GenBank accession numbers NC003604, NC003602) and Israel (AF007515, EF583906) (Sardarelli et al. 2000; Galiakparov et al. 1999; Moskowitz et al. 2008), they represent only a small part of the extensive genetic heterogeneity of these viruses (Fig. 1). Recent results suggest that the variants of these viruses vary in pathogenicity to grapevines (Goszczynski and Jooste 2003b; Goszczynski 2007, 2010). In addition, the clones constructed in Italy were highly unstable, which eliminated them from further applications (Sardarelli et al. 2000). The cause of the instability of the clones was unknown. For the construction of DNA clones reported in this paper, genetic variants GVA P163-M5 and GVB 953-1, sharing only 76.3 %, 73.2 % and 85.2 %, 77.6 % nt genome similarity with the corresponding viruses from Italy and Israel, were used (Fig. 1). The full genome sequences of these variants are deposited in GenBank under accession numbers DQ855082 and KJ524452, respectively. The GVA variant P163-M5 was isolated in *Nicotiana benthamiana* from grapevine cv. Cinsaut Blanc clone P163/12 (Goszczynski and Jooste 2003a, b; Goszczynski et al. 2008). The grapevine was used by the South African grapevine industry as a very reliable source of SD in woody indexing. In our laboratory, the disease was also easily graft transmitted from C. Blanc clone P163/12 to SD-susceptible Shiraz and Merlot, on several occasions. In some cases the transmission was successful even when the graft had not taken (Goszczynski, not published). This indicates the presence of an exceptionally highly virulent pathogen in this grapevine that causes SD. In addition to GVA, this grapevine is infected with *Grapevine rupestris stem pitting*-*associated virus* (GRSPaV) and *Grapevine leafroll*-*associated virus* 3 (GLRaV-3) (Goszczynski, not published). The GVA population consists of two genetic variants, P163-M5 and P163-1, which are highly divergent, sharing only 70.8 % nt genome similarity. The variant P163-M5 is a member of phylogenetic group II associated with SD in South Africa (Goszczynski and Jooste 2003a; Goszczynski et al. 2008). Variants of this group are also commonly present in grapevines affected by Australian Shiraz disease (AuSD) (Goszczynski and Habili 2012). The variant P163-M5 is unique among variants of group II as it has 119 nt insert in ORF2. The insert sequence share 75.6 % nt similarity with the corresponding native sequence of this variant, and 68.1–78.2 % nt similarity with other members of the group II identified in South Africa (Goszczynski et al. 2008). The GVB variant 953-1 was isolated in *N. benthamiana* from grapevine LN33 hybrid, our grapevine collection accession number 93/953, exhibiting clear cane symptoms of CB disease (Goszczynski 2010). The grapevine, in addition to GVB, is also infected with GRSPaV and GLRaV-3 (Goszczynski, not published). The population of GVB consists of two divergent variants of the virus, 953-1 and H1, sharing 78 % nt genome similarity (Goszczynski 2010). The variant GVB H1 was also detected as a single infection in the LN33 hybrid, which consistently and over years, does not exhibit CB symptoms (Goszczynski 2010). It suggests that the GVB variant 953-1 may be responsible for CB symptoms in LN33 93/953, and this makes it the perfect candidate for the construction of a DNA clone.

## Methods

**Fig. 2:**
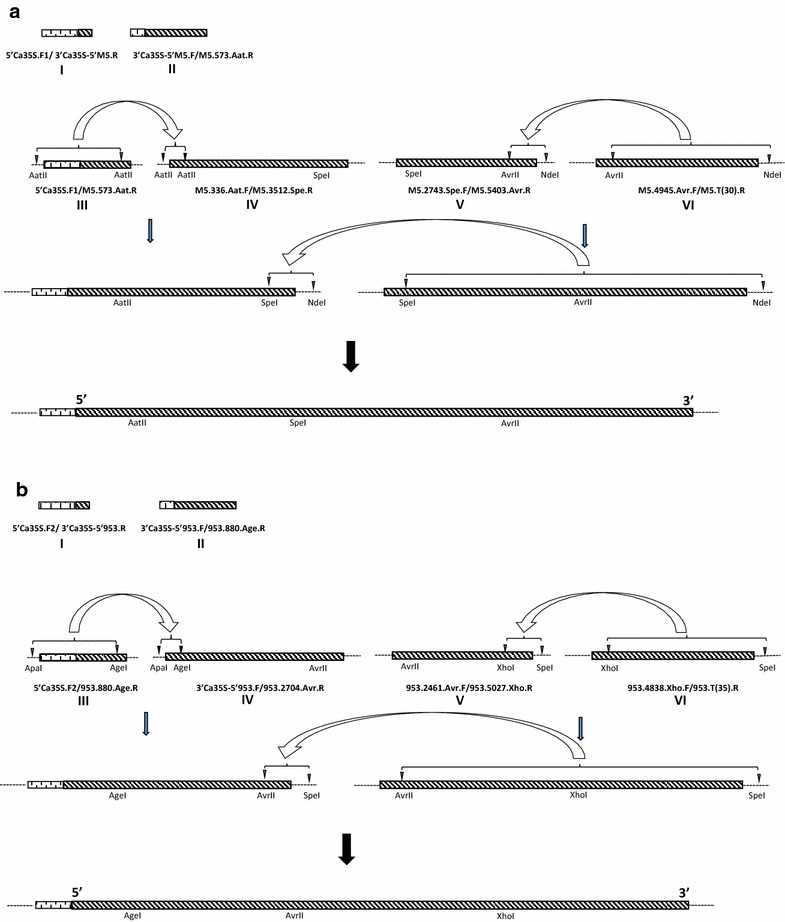
General strategy of construction of DNA clones of **a** GVA and **b** GVB genetic variants P163-M5 and 953-1, respectively. Numbers I–VI describe RT-PCR amplified CaMV 35S promoter and virus genome fragments using primers which nucleotide sequences are shown it Table 1. The fragments III-VI were cloned into the TA site of pGEM-T Easy vector (Promega) (*single dashed line*) and then assembled in this vector using carefully selected restriction enzymes to digest the vector and virus sequences. The* open arrows* show the sequence of the assembling of virus fragments in the vector. *Closed arrows* show the stages of ligation of virus fragments to obtain full-length DNA clones of viruses under CaMV 35S promoter

A standard approach was applied in constructing DNA clones of GVA and GVB. The replicative form (RF) of dsRNA was used as template in RT-PCR amplifications of the virus genome sequence. The total dsRNA was isolated from symptomatic leaves of virus-infected *N. benthamiana* according to Valverde et al. (1990). RF was separated from this dsRNA by electrophoresis in agarose, and then extracted from gel using Zymoclean™ Gel RNA Recovery Kit (Zymo Research). In single RT-PCR amplification RFs of GVA and GVB purified from about 250 mg of virus-infected *N. benthamiana* were used. The GVA and GVB genomes were RT-PCR amplified in four overlapping 592–3176 bp DNA fragments containing unique restriction sides in overlapping regions (Fig. 2). Expand reverse transcriptase and Expand long range DNTP pack kits, both from Roche, were used. The cauliflower mosaic virus (CaMV) Ca35S promoter was amplified from binary plasmid pCAMBIA1305.1, and linked to DNA fragment complementary to the 5′ end of the virus genome in PCR described by Peremyslov and Dolja (2007). The oligonucleotide virus-specific and Ca35S-specific primers sequence used in this study were designed manually using the GenBank sequence data, and are shown in Table 1. The RT-PCR-amplified fragments were cloned into the TA site of pGEM-T Easy vector (Promega) and transformed to highly competent cells of *Escherichia coli* 5α (Zymo Research). The cloned virus genome fragments were digested with restriction enzymes, purified from agarose and re-assembled to the full GVA and GVB genome in pGEM-T Easy vector using T4 ligase (Thermo Scientific) as shown in Fig. 2. To minimise the chances of lethal mutations, which could be introduced by RT-PCR, 6–10 clones of sequences directly amplified from dsRNA of viruses were combined before proceeding to the next step.

**Table 1:** Oligonucleotide primers used in the construction of DNA clones of GVA and GVB genetic variants P163-M5 and 953-1, respectively, shown in Fig. 2

Virus	Primer code	Sequence (5′–3′)	Amplified fragment^a^
GVA P163-M5	5′Ca35S.F1	GGATCC**CATGGAGTCAAAGATTC**	
	3′Ca35S-5′M5.R	GGGAATCAAGTTAAATATTCG**AGTCCCCCGTGTTCTCTCC**	I
	3′Ca35S-5′M5.F	**GGAGAGAACACGGGGGACT**CGAATATTTAACTTGATTCC	
	M5.573. Aat.R	GCATTGGTGCTTATCCTC	II
	M5.336.Aat.F	CACATGCTCTATAGAGTTGCACC	
	M5.3512.Spe.R	CTTGACCTGGTTAGAGCGA	IV
	M5.2743.Spe.F	AGGCTGACAGACTGGCGCGGAGC	
	M5.5403.Avr.R	CACTCAACGTAGGACAAGGACTC	V
	M5.4945.Avr.F	ATGGCAAAGAGGAACGG	
	M5.T(30).R	CATATGT(30)GTCATAGTATGACAACCTAGC	VI
GVB 953-1	5′Ca35S.F2	ATGCGGCCGC**CATGGAGTCAAAGATTC**	
	3′Ca35S-5′953.R	CGGAAGATTAAAGAAAAAGVGAACTTTATTG**AGTCCCCCGTGTTCTCTCC**	I
	3′Ca35S-5′953.F	**GGAGAGAACACGGGGGACT**CAATAAAGTTCGCTTTTTCTTTAATCTTCCG	
	953.880.Age.R	ATGTGCTCCCTCTGGTGGCC	II
	953.2704.Avr.R	TGGCTCTTTGCTCGCACACC	IV^b^
	953.2461.Avr.F	AGGGGTGGTATGACACTTGG	
	953.5027.Xho.R	CTAACCAGATCCTGCTGAGC	V
	953.4838.Xho.F	CGAGGATGAGCCCCTATGGC	
	953.T(35).R	AGTTCGACT(35)TCGTGTTTATTCACGCTTCTTTCACC	VI

In bold are shown parts of the sequence of the CaMV 35S promoter in various orientations

^a^See Fig. 2. Fragments III of GVA P163-M5 and GVB 953-1 were PCR amplified using fragments I and II as templates, and primers pair 5′Ca35S.F1/M5.573.Aat.R and 5′Ca35S.F2/953.880.Age.R, respectively, according to the method described by Peremyslov and Dolja (2007)

^b^Fragment IV of GVB 953-1 was amplified using primers pair 3′Ca35S-5′953.F/953.2704.Avr.R

The full DNA copies of virus variants under Ca35S promoter that were obtained in pGEM-T vector were released from this plasmid by digestion with selected restriction enzymes (Fig. 2), blinded with T4 DNA polymerase (Thermo Scientific) and cloned to binary vector pCAMBIA1305.1 (11,846 bp) and later to modified binary vector pCB302-NoX-ER-CFP (Peremyslov and Dolja 2007). The pCB302-NoX-ER-CFP vector (6956 bp) kindly donated by Dr. V. Peremyslov (Oregon State University, USA) is a derivative of the mini binary vector pCB302 reported by Xiang et al. (1999). The vector was made by adding an expression bar cassette composed of a Ca35S promoter and Ca35S terminator and the ER-CFP reporter to pCB302 sequence. The modification of pCB302-Nox-ER-CFP was done by removing 1704 bp part of the expression bar cassette between *Xba*I and *Eco*RI sites, leaving Ca35S terminator and Nos terminator on both ends of the linearised vector. This vector is named here as pCB.LB.35ST-NosT.RB. EcoRI digested pCAMBIA 1305.1 (11,846 bp) and *Xba*I/*Eco*RI digested pCB.LB.35ST-NosT.RB (5252 bp) binary vectors were blinded and de-phosphorylated, using respectively T4 DNA polymerase and ATP phosphatase (Thermo Scientific) before ligation with blinded full cDNA copies of the viruses, using T4 DNA ligase. In each cloning, the presence of the plasmids with correct, partial or full DNA copies of the variants sequences was monitored using predicted *Eco*RI and *Xba*I digestion patterns as a guide, respectively.

Plasmids purified from selected 10 *E. coli* containing full DNA copies of viruses under Ca35S promoter were mixed, and the mixture was used to transform *Agrobacterium tumefaciens* C58 C1. Competent cells of *A. tumefaciens* C58 C1 were prepared according to the procedure (mini-scale) published on line by Tsai and Harding (2008). *E. coli* (see above) and *A. tumefaciens* colonies containing binary vectors with DNA copies of GVA P163-M5 and GVB 953-1 were identified by PCR using virus-specific primer pairs M5.2743.Spe.F/M5.3512.Spe.R and 953.2461.Avr.F/953.2704.Avr.R (Table 1). The randomly selected individual clones of *A. tumefaciens* C58.C1 that were PCR-positive for virus sequences were cultured in LB medium with antibiotics (Rifampicin, 25 µg/ml; Tetracycline, 5 µg/ml; Kanamycin, 50 µg/ml) overnight at about 30 °C with vigorous shaking, then 1.8 ml of bacterial culture was pelleted by centrifugation 5000 rpm for 10 min at 20 °C, and re-suspended in 0.4 ml of inoculation medium (10 mM MES, 10 mM MgCl_2_ and 150 µM acetosyringone). After an incubation of 2 h at room temperature in the dark, the bacteria were injected to the slightly wounded underside of leaves of *N. benthamiana* plants (at 6–8 leaf stage) using a 1 ml syringe without needle. Agroinoculated plants were kept in a growth room at 25–27 °C, under a 12 h light cycle.

Purification of total dsRNA from virus-infected symptomatic *N. benthamiana* was carried out essentially as described by Valverde et al. (1990), using two rounds of dsRNA purification through CF11 cellulose. For pattern analysis of virus-specific dsRNA the samples of total dsRNA corresponding to about 300 mg of virus-infected tissue of *N. benthamiana* were electrophoresed in 6 % polyacrylamide gels at 100 V for 3 h at +5 °C, using Mini-protean II dual slab cell (Bio-Rad).

Western Blot analysis of virus capsid proteins was done as reported by Goszczynski et al. (1997). The proteins were extracted from symptomatic leaves in 0.1 M Tris–HCl buffer pH 7.6 containing 2.5 % SDS, 5 % glycerol and 5 % 2-mercaptoethanol, 2.0 ml per 0.5 g of tissue, then incubated in boiling water for 2.5 min and centrifuged at 14,000 rpm for 2 min. The supernatant was stored frozen at −80 °C and used, without further processing, for separation of virus capsid proteins in mini SDS-PAGE gels. To detect the virus capsid proteins, the virus-specific rabbit antisera produced earlier to capsid proteins of GVA isolate 92/778 (Goszczynski and Jooste 2003a) (Goszczynski, not published) and GVB isolate 94/971 purified from SDS-PAGE gels (Goszczynski et al. 1997) were used. The GVA and GVB-specific rabbit antisera and goat anti rabbit alkaline phosphatase conjugate (GAR-AP) were used diluted 1000 and 2000 times respectively.

## Results and discussion

Full genome DNA copies of both GVA P163-M5 and GVB 953-1 under 35S promoter cloned into pGEM-T vector could be easily found among transformants of *E. coli*. However, after re-cloning to the binary vector pCAMBIA 1305.1 this applied only to the DNA clone of GVA P163-M5. For the DNA clone of this variant, among 65 colonies of *E. coli* tested by PCR, 17 were positive for the virus, and 5 had correct predicted EcoRI pattern for full DNA copy of the variant genome. On the contrary, for DNA clone GVB 953-1, testing of total 229 *E. coli* colonies, 28 were found virus-positive in PCR, and only 4 had the correct, predicted XbaI pattern of the full DNA copy of the variant. Purified plasmids containing full DNA copies of GVA P163-M5 and GVB 953-1, respectively, under Ca35S promoter were combined and transformed to *A. tumefaciens* C58.C1. Fourteen PCR virus-positive colonies of each virus were selected for agroinoculation of *N. benthamiana*. Of these, 8 DNA clones of GVA P163-M5 and none of GVB 953-1 were infectious to *N. benthamiana*. To uncover the reason for the lack of infectivity of DNA clones of GVB 953-1, the mixture of the full DNA copies of this virus under Ca35S promoter released from pGEM-T vector, used earlier in cloning to pCAMBIA 1305.1, stored in −30 °C, was used to clone the virus to pCB.LB.35ST-NosT.RB. The same was done with a similar mixture of the full DNA copies of GVA P163-M5. For each virus, among 10 randomly selected *E. coli* colonies which were PCR positive for virus sequences, 6 and 8 colonies contained full DNA copies of GVA P163-M5 and GVB 953-1, respectively. Plasmids with full DNA copies of viruses under Ca35S promoter were mixed and transformed to *A. tumefaciens* C58.C1. Brief PCR testing of respectively 4 and 25 *A. tumefaciens* colonies, 2 and 5 were virus-positive. The positive clones were used to agroinoculate *N. benthamiana*. Of these, 2 DNA clones of GVA P163-M5 and 4 DNA clones of GVB 953-1 were infectious. These results suggest that the lack of infectivity of DNA clones of GVB 953-1 variant in pCAMBIA1305.1 observed earlier, was caused not by lethal mutations, which could be introduced to virus genome in RT-PCR or toxicity of the variant to *E. coli*, but was apparently caused by instability of the DNA copy of this variant under Ca35S promoter in this binary vector. The relatively easy cloning of infectious DNA copies of both GVA and GVB to pCB.LB.35ST-NosT.RB, suggest that this binary vector is superior to pCAMBIA1305.1 in cloning of the full cDNA copies of members of the *Vitivirus* genus.

**Fig. 3:**
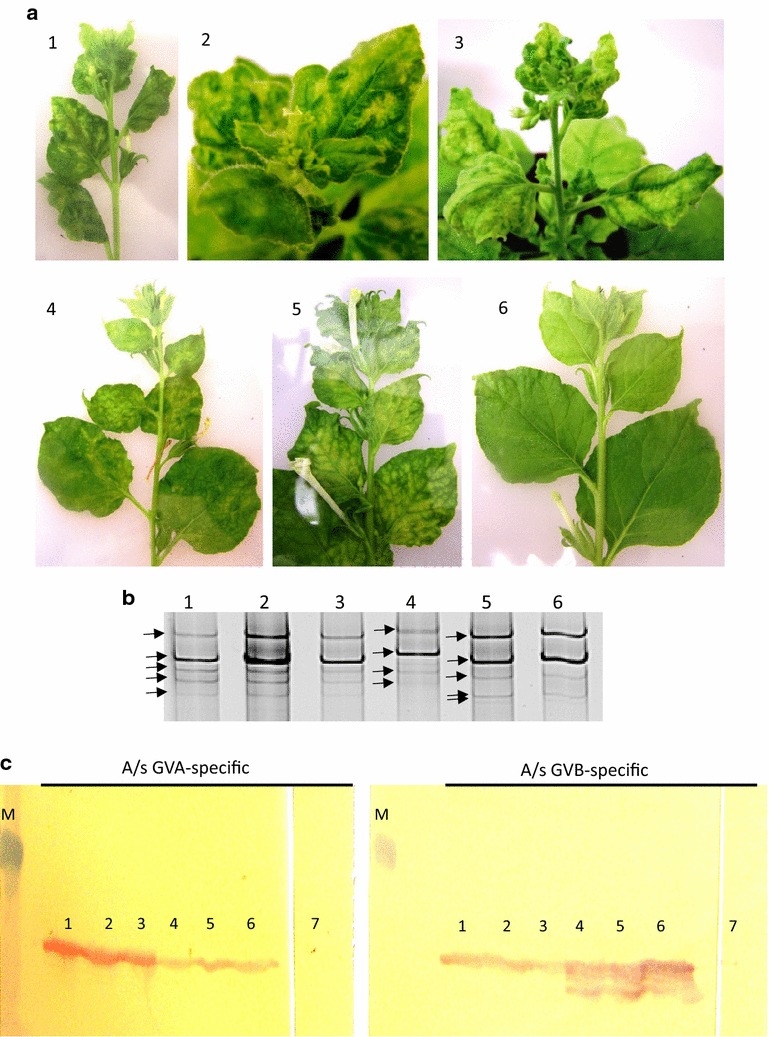
**a** Systemic symptoms (30 dpi) induced in leaves of *N. benthamiana* inoculated with DNA clones of (1, 2) GVA and (4) GVB genetic variants P163-M5 and 953-1, and the wild-type of these variants (3, 5), respectively. (6) Virus-free *N. benthamiana.* **b** dsRNA extracted from systemically virus infected symptomatic leaves of *N. benthamiana* inoculated with (1–3) GVA and (5, 6) GVB genetic variants P163-M5 and 953-1, using (1, 2, 5) DNA clones and (3, 6) the wild-type of these variants, respectively. *Lane* 4 shows dsRNA extracted from *N. benthamiana* inoculated with the mild wild-type GVA, variant P163-1 (Goszczynski and Jooste 2003a). *Arrows* point to dsRNA fragments clearly visible in EtBr-stained 6 % polyacrylamide gels. **c** Western Blot reaction of GVA- and GVB-specific rabbit antisera, produced to capsid proteins of these viruses (Galiakparov et al. 2003), with extracts from (1–6) virus-infected *N. benthamiana* inoculated with DNA clones of (1, 2) GVA and (4, 5) GVB genetic variants P163-M5 and 953-1, and (3, 6) the wild-type of these variants, respectively. *Lanes* 7 show lack of reaction of these antisera with extracts from virus-free plants. (M) Prestained MW marker (38 kDa, Sigma). Virus-specific rabbit antibodies were detected by incubation of nitrocellulose membranes with goat anti-rabbit alkaline phosphatase conjugate (GAR-AP) (Sigma). The mixture of Naphthol AS-MX phospohate (Sigma) and Fast Red TR salt (Sigma) in 0.2 M Tris–HCl buffer, pH 8.2, was used as substrate for the alkaline phosphatase (Goszczynski et al. 1997)

*Nicotiana benthamiana* agroinoculated with *A. tumefaciens* containing the full DNA copy of GVA P163-M5 under Ca35S promoter, cloned to binary vectors pCAMBIA1305.1 as well as to pCB.LB.35ST-NosT.RB, exhibited systemic symptoms of virus infection after about 1 week. The symptoms of vein clearing, yellowing, and severe deformation of leaves, were similar to symptoms induced by the wild type of this GVA variant in this host (Fig. 3a1–3). In the case of DNA clones of GVB 953-1, in pCB.LB.35ST-NosT.RB binary vector, the systemic symptoms of yellow mottling appeared about 2 weeks after agroinoculation. The symptoms were also similar to symptoms induced by the wild type of this variant (Fig. 3a4, 5). To test the stability of DNA clones of GVA P163-M5 and GVB 953-1 the clones were passaged 5 times on LB medium with antibiotics and used for agroinoculation of *N. benthamiana*. Each time the clones induced the same symptoms in this herbaceous host, indicating that they were stable. Polyacrylamide gel electrophoresis of virus dsRNA extracted from symptomatic leaves of *N. benthamiana* revealed the same dsRNA pattern as the wild type of variants (Fig. 3b). Also, Western Blot analysis of virus capsid proteins extracted from these plants had the similar electrophoretic mobility as capsid proteins of the wild type of variants and clearly reacted with the virus-specific rabbit antisera produced to capsid proteins of wild-type viruses purified from SDS–polyacrylamide gels (Fig. 3c). The cross-reaction of the antisera visible in Fig. 3c was due to the fact that the viruses are serologically related (Goszczynski et al. 1997). As the antisera did not react with the extracts from virus-free *N. benthamiana*, the multiple bands visible on blots treated with GVB-specific antiserum (Fig. 3c, lanes 4–6) revealed the reaction of the antiserum with the partially degraded capsid protein of GVB. The same effect was observed in blots treated with this GVB-specific antiserum earlier (Goszczynski et al. 1996), and suggests that the capsid protein of GVB is unstable in plant extracts.

The DNA clones of GVA P163-M5 and GVB 953-1 reported here will be used to investigate the aetiology of SD and CB diseases. As the clones were constructed using virus dsRNA isolated from *N. benthamiana*, to avoid the possibility that they will not be infectious to grapevines due to adaptation to this herbaceous host (Kurth et al. 2012), construction of clones of the same variants using dsRNA of viruses isolated directly from grapevines is underway. The clones will also have other applications. They will be used to investigate of the still mysterious function of a protein encoded by ORF2 of the members of the *Vitivirus* genus. Although it was determined that this protein is dispensable for the biology of GVA in *N. benthamiana* (Galiakparov et al. 2003), nothing is known about the function of this protein in the grapevine host. As was mentioned, the DNA clone of GVA variant P163-M5 has unique ORF2 among variants of this virus. In addition, as GVA and GVB infections are latent in most grapevine cultivars, the DNA clones of these viruses can be used as vectors of grapevine sequences in analysis of the grapevine genome using virus-induced gene silencing (VIGS) (Muruganantham et al. 2009).

## Conclusion

Reported here stable DNA clones of GVA and GVB enrich the pool of DNA clones of these extensively genetically heterogenic and relatively unknown viruses. The clones may substantially contribute in the further study of these viruses.
